# Supplementary material for: Steamed Multigrain Bread Prepared from Dough Fermented with Lactic Acid Bacteria and Its Effect on Type 2 Diabetes
Source: Foods. 2023 Jun 9;12(12):2319. doi: 10.3390/foods12122319 (PMC10297133; doi:10.3390/foods12122319)
Supplement: Supplementary file 1 [file foods-12-02319-s001.zip › foods-2389260-supplementary.pdf]

Supporting Information

MSB FMSB WSB

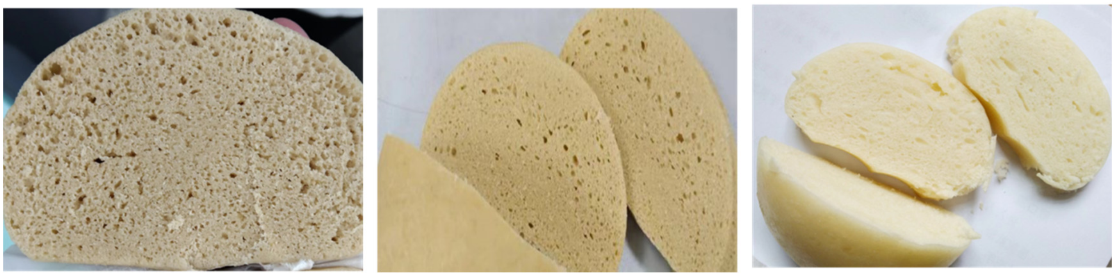

Figure S1 Characteristics of steamed bread section. MSB, steamed multigrain bread without lactic acid bacteria; FMSB, steamed multigrain bread with lactic acid bacteria; WSB, steamed wheat flour bread.
